# Epidemiology of *Haemophilus parasuis* isolates from pigs in China using serotyping, antimicrobial susceptibility, biofilm formation and ERIC-PCR genotyping

**DOI:** 10.7717/peerj.5040

**Published:** 2018-06-13

**Authors:** Yongda Zhao, Qin Wang, Jie Li, Xiaohuan Lin, Xianhui Huang, Binghu Fang

**Affiliations:** College of Veterinary Medicine, South China Agricultural University, Guangzhou, Guangdong, China

**Keywords:** Biofilm formation, *Haemophilus* parasuis, ERIC-PCR, Serotype, Antimicrobial susceptibility

## Abstract

**Background:**

*Haemophilus parasuis* is a commensal organism of the upper respiratory tract of healthy pigs and causes high morbidity and mortality in piglets. The aim of this study was to investigate the epidemiology of *H. parasuis* in China from 2014 to 2017.

**Methods:**

We characterized 143 *H. parasuis* isolates by serotyping, antimicrobial susceptibility, biofilm formation and with enterobacterial repetitive intergenic consensus-polymerase chain reaction (ERIC-PCR) assays.

**Results:**

Serotyping revealed serovar 5 as the most prevalent (26.6%) followed by serovars 4 (22.4%), 7 (9.1 %), 13 (6.3%), 12 (5.6 %), and non-typeable (8.4%). In a panel of 23 antimicrobials, the minimum inhibitory concentration 50% (MIC_50_) were in the range of 0.25–16 μg/mL and MIC_90_ were 2–>512 μg/mL. A total of 99 isolates of *H. parasuis* (69.2%) were able to form biofilms and 59.6% (59/99) performed weak biofilm-forming ability. ERIC-PCR revealed a very heterogeneous pattern with 87 clusters.

**Discussion:**

These *H. parasuis* isolates showed a high serovar and genotypic lineage diversity, different abilities to form biofilms and a high degree of genetic diversity. Biofilm formation was related to antimicrobial susceptibility but there were no statistically significant associations between the antimicrobial susceptibility and either the serovars or the ERIC-PCR clusters. This study showed a high prevalence of high-MIC *H. parasuis* strains and suggests the need for a continuous surveillance of clinical isolates of *H. parasuis*.

## Introduction

The Gram-negative bacterium *Haemophilus parasuis* is the causative agent of Glässer’s disease in pigs and is one of the most important pathogen in the modern swine industry. The disease is characterized by pneumonia, meningitis, arthritis, polyserositis, and septicemia. Outbreaks of Glässer’s disease have resulted in serious lethality and economic losses in the global swine industry (Kim et al., 2002; Oliveira, Blackall & Pijoan, 2003; Palzer et al., 2015; Wang et al., 2017; Yu et al., 2012). There are 15 serovars of *H. parasuis* but also a large number of non-typeable (NT) isolates and these possess wide differences in virulence (Kielstein & Rapp-Gabrielson, 1992). However, serovar diversity has hampered effective cross-protection using current vaccines resulting in antimicrobial treatments as the first line of defense against the disease (Oliveira & Pijoan, 2004).

A range of antimicrobial agents including quinolones, β-lactams, macrolides, phenicols, sulfonamides and tetracyclines have been administered in feed, water or by parenteral administration for the treatment and prevention of respiratory infections caused by *H. parasuis*. Some of these antimicrobials have been given at sub-therapeutic dosages to promote growth and aid in the prevention of disease. But the extended use of antimicrobials has facilitated the emergence and development of antimicrobial resistance and even multidrug resistance in this organism (Aarestrup et al., 2004; de la Fuente et al., 2007; Karriker et al., 2013).

Bacterial biofilms are membrane-like bacterial clusters that attach to living or inert objects and are formed by bacterial reproduction, differentiation, and secretion of polysaccharide matrices (Donlan & Costerton, 2002). Bacteria within biofilms escape the killing effects of antimicrobials and can resist attacks of the host immune system (Hall-Stoodley, Costerton & Stoodley, 2004; Jacques, Aragon & Tremblay, 2010). Most *H. parasuis* serovars are capable of in vitro biofilm formation and the expression of genes with putative functions in biofilm formation have been detected during pulmonary infections (Jin et al., 2008). Biofilm formers have greater antimicrobial resistance especially for β-lactam antimicrobials (Zhang et al., 2014). Biofilm formation might therefore allow the non-virulent strains to colonize and persist in the upper respiratory tract of pigs (Bello-Orti et al., 2014). Therefore, biofilms are an important part of the infection process for this bacterial pathogen.

Nevertheless, since 10–60% of the isolates are NT, serotyping is not discriminative enough to enable epidemiologic studies to guide vaccine development (Moreno et al., 2011; Oliveira & Pijoan, 2004; Rapp-Gabrielson, Oliveira & Pijoan, 2006). For this reason, molecular methods have been developed to improve diagnostic and epidemiological characterization of *H. parasuis*. Among them, enterobacterial repetitive intergenic consensus-polymerase chain reaction (ERIC-PCR) is a genotyping method based on conjugated enterobacterial repeat sequences. It is a valuable tool for typing *H. parasuis* isolates including the serologically NT ones in a rapid and low cost manner (Moreno et al., 2011). The discriminatory power of ERIC-PCR has been successful in epidemiological studies of *H. parasuis* (Macedo et al., 2011).

The knowledge of serovar distribution, antimicrobial susceptibility, biofilm formation, and genotypes of *H. parasuis* will help to understand the current state of *H. parasuis* epidemiology, and may be useful the control of Glässer’s disease in China. The objective of this study was to characterize 143 *H. parasuis* isolates recovered from clinical cases by serotyping, antimicrobial susceptibility testing, biofilm formation, and ERIC-PCR genotyping.

## Materials and Methods

### Serotyping

**Figure 1:**
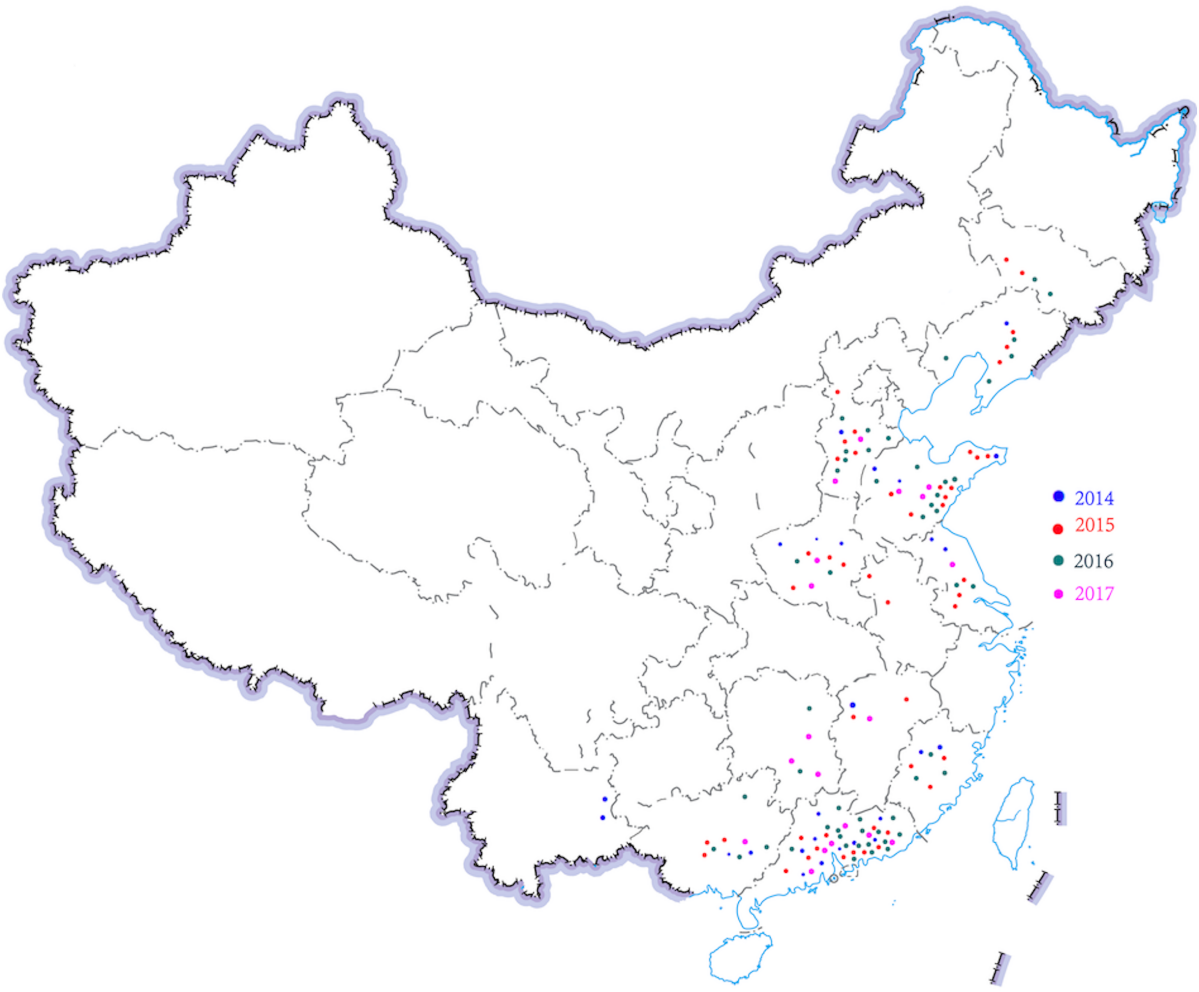
Region distribution of 143 *H. parasuis* strains in China from 2014 to 2017. Distribution of different time and region of *H. parasuis* in northeast, north, east, central, south, and southwest China.

A total of 143 *H. parasuis* strains were studied (Table 1; Fig. 1). The information of the 143 *H. parasuis* strains has been reported (Zhao et al., 2018). The South China Agriculture University Animal ethics committee approved to carry out the study (No. 2014-025). Serotypes of all 143 *H. parasuis* field isolates were identified using the gel diffusion (GD) test against 15 reference strain antiserums as previously described (Morozumi & Nicolet, 1986; Turni & Blackall, 2005) and the GD methodology, subjected to indirect hemagglutination (IHA) testing (Turni & Blackall, 2005). Non-typed strains were defined as isolates whose antigens did not react with antiserum against the 15 reference serotypes.

**Table 1:** Isolation time and region of 143 *H. parasuis* isolates.

Year	Number	Ratio (%)	Region of China					
			Northeast	North	East	Central	South	Southwest
2014	26	18.18	1	1	9	4	9	2
2015	47	32.87	5	5	19	4	14	0
2016	51	35.66	6	7	13	5	19	1
2017	19	13.29	0	3	6	4	6	0

**Note:**

Number of strains of *H. parasuis* isolates from different region of different year from 2014 to 2017.

### Antimicrobial susceptibility testing

The minimum inhibitory concentration (MIC) values of antimicrobials for the *H. parasuis* were determined by a micro double-dilution method according to protocols by the Clinical and Laboratory Standards Institute (CLSI, 2013) and a previous report (Pruller et al., 2017). The 23 antimicrobial agents used for susceptibility testing were amoxicillin, ampicillin, cephalexin, cefepime, ceftiofur, ciprofloxacin, doxycycline, enrofloxacin, florfenicol, gamithromycin, gentamicin, kanamycin, lincomycin, lomefloxacin, oxacillin, polymyxin B, penicillin, spectinomycin, tulathromycin, tildipirosin, tetracycline, tilmicosin, trimethoprim/sulfamethoxazole. The National Institute for the Control of Pharmaceutical and Biological Products in Beijing, China supplied all antimicrobials. Cation-adjusted Mueller–Hinton broth (Becton Dickinson, Owings Mills, MD, USA), nicotinamide adenine dinucleotide (NAD) (Sigma, St. Louis, MO, USA) and chicken serum (Ruite, Guangzhou, China) were used for MICs detection. Performance and evaluation of MIC determinations followed the standards of the Clinical and Laboratory Standards Institute (CLSI) Table 2 *H. influenzae* and *H. parainfluenzae* M02 and M07 (CLSI, 2015).

**Table 2:** Serotype distribution of 143 *H. parasuis* isolates for different region.

Serotype	Different region of China (*n* = 143)						Total	Percent (%)
	Northeast	North	East	Central	South	Southwest		
1	1		2		3		6	4.2
2			2				2	1.4
3			2	1			3	2.1
4	3		10	4	15		32	22.4
5	3	5	13	6	11		38	26.6
6	1	2	1		1		5	3.5
7	2	2	4	1	3	1	13	9.1
8			1		1		2	1.4
9			1				1	0.7
10		1	1				2	1.4
11		1			1		2	1.4
12		1	1	2	3	1	8	5.6
13	1	1	4		3		9	6.3
14	1		2	1	2		6	4.2
15					1	1	2	1.4
NT		3	3	2	4		12	8.4
Total	12	16	47	17	48	3	143	1
Percent(%)	8.4	11.2	32.9	11.9	33.6	2.1	1	/

**Note:**

The percent in the last line is calculated as different region. The percent in the right side column is calculated as different serovars.

Ranges of susceptibility were performed varying from 0.25 to 512 μg/mL of the antibiotic described above and recorded along with the MIC that inhibited growth of 50% (MIC_50_) and 90% (MIC_90_) of the isolates. The reference strains *H. influenzae* ATCC 49247 and *Escherichia coli* ATCC25922 served as quality controls for MIC determinations.

### Biofilm formation

Biofilm formation experiments were performed in 96-well microtiter plates as previously described with minor modifications (Jin et al., 2006; Stepanovic et al., 2000). Briefly, all *H. parasuis* isolates from overnight agar cultures were suspended in 5 mL tryptic soy broth (TSB) (Becton Dickinson, Owings Mills, MD, USA) containing 10 μg/mL NAD and 5% bovine serum (Gibco®, Auckland, New Zealand) and 200 μL of this culture suspension was aliquoted in triplicate into flat-bottom 96 well polystyrene plates and incubated for 24 h at 37 °C. The wells were washed three times with 200 μL of sterile phosphate-buffered saline to remove loosely adherent cells. The remaining attached bacteria were fixed with 200 μL of absolute methanol per well for 15 min before the plates were emptied and left to dry at room temperature. Biofilms were stained with 200 μL of 1% (w/v) crystal violet for 5 min. Excess stain was removed with three washes of distilled water and the plates were dried at 37 °C for 15 min. The stain was then released by adding 100 μL of 33% (v/v) glacial acetic acid per well. The amount of released stain was quantified by measuring the absorbance at 570 nm with an automated Elx800 Universal Microplate Reader (Bio-Tek Instruments Inc., Winooski, VT, USA).

### Molecular typing by ERIC-PCR

*Haemophilus parasuis* isolates were grown on tryptic soy agar (Becton Dickinson, Owings Mills, MD, USA) plates and several colonies of each isolate were inoculated into 5 mL TSB containing 10 μg/mL NAD and 5% bovine serum and incubated for 24 h at 37 °C. Total DNA was extracted from these cultures using the TIANamp Bacteria DNA Kit (Tiangen Biotech, Beijing, China). The isolates were characterized by ERIC-PCR following published guidelines (Rafiee et al., 2000). PCR reactions were carried out with the following cycling conditions: degeneration 5 min at 94 °C, 35 cycles of 30 s at 94 °C, 60 sec at 50 °C and 2 min at 72 °C and a final step of 10 min at 72 °C. The resulting band profiles were visually assessed using BioNumerics 6.6 software. A 90% similarity cut-off was used to analyze the genotypes generated by ERIC-PCR technique according to Oliveira, Blackall & Pijoan (2003).

## Results

### Serotyping

*Haemophilus parasuis* strains (143) were serotyped using GD and IHA tests and 131 were typeable. The serotype prevalence was serotype 5 (26.6%), 4 (22.4%), 7 (9.1%), NT (8.4%), 13 (6.3%), 12 (5.6 %), 1 (4.2%), 14 (4.2%), and 6 (3.5%). In addition, there were also some serotypes that existed only in one or two strains such as serotype 3 (2.1%), 2 (1.4%), 8 (1.4%), 10 (1.4%), 11 (1.4%), and 9 (0.7%) (Table 2).

**Figure 2:**
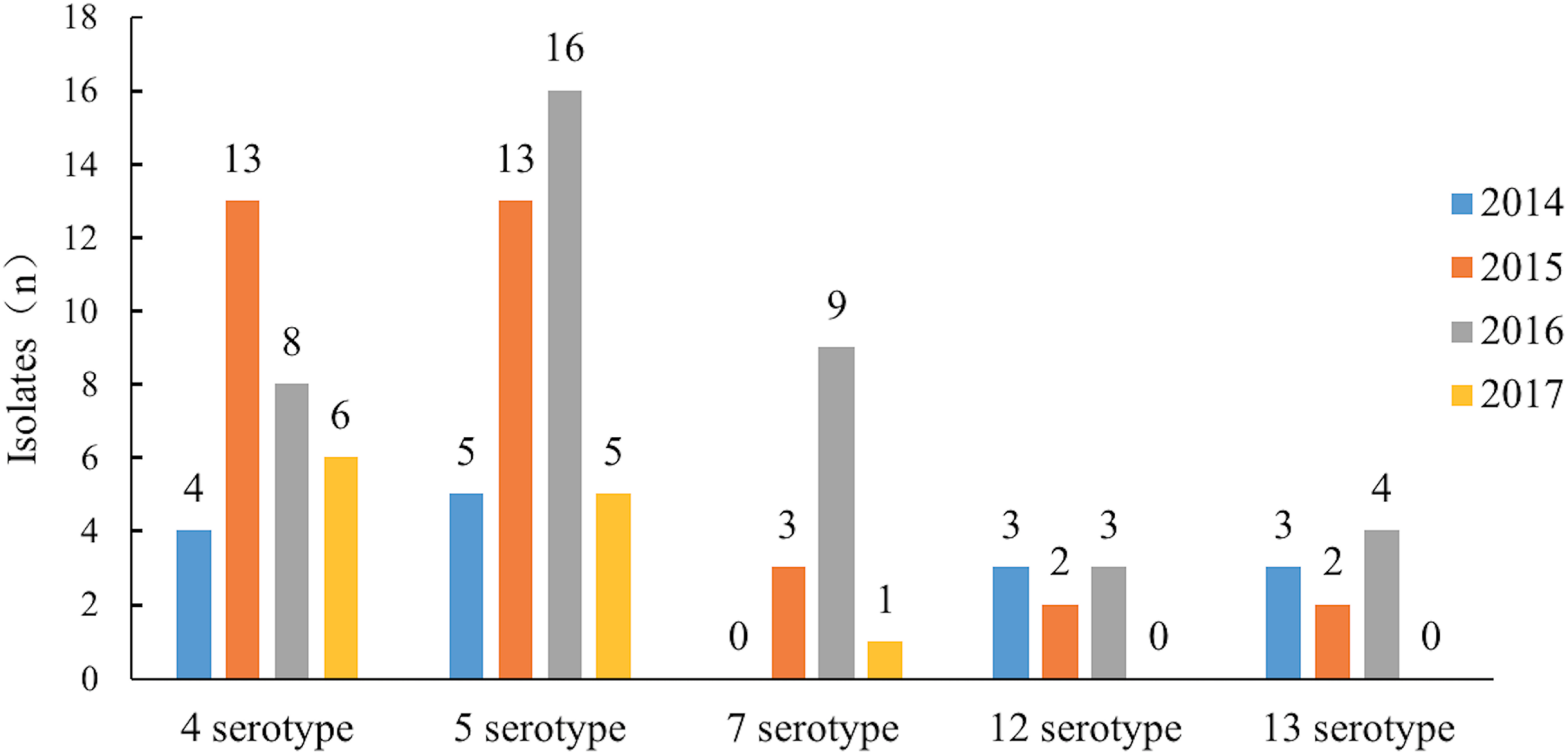
The epidemic trend of the major serotypes from 2014 to 2017.

Serotypes 4 and 5 were the major serotypes associated with the most recent epidemics in China. In 2104 and 2015, serotypes 12 and 13 were the main serotypes after serotype 4 and 5 but after 2016, the number of isolates of serotype 7 increased rapidly, significantly beyond serotypes 12 and 13. Serotype 7 became one of the major epidemic serotypes (Table 2; Fig. 2).

### Antimicrobial susceptibility testing

Minimum inhibitory concentration values for the reference strains were within the acceptable quality control ranges (CLSI, 2015). The results of the susceptibility testing of the 23 antimicrobials against 143 isolates of *H. parasuis* (See Supplemental Material S1) were tabulated as distribution of the MIC values, MIC_50_, MIC_90_. The susceptibility tests indicated that the MIC_50_ values of all 23 tested antimicrobials were in the range of 0.25–16 μg/mL and MIC_90_ values were 2–>512 μg/mL (Table 3).

**Table 3:** Antimicrobial susceptibility of 143 *H. parasuis* isolates from China 2014 to 2017.

Antimicrobials	Number of *H. parasuis* strains with MICs (μg/mL)														MIC_50_ (μg/mL)	MIC_90_ (μg/mL)
	<0.25	0.25	0.5	1	2	4	8	16	32	64	128	256	512	>512		
Amoxicillin	0	61	17	22	8	5	4	14	4	6	2	0	0	0	0.5	16
Ampicillin	1	3	47	30	18	9	7	8	2	4	0	3	7	4	1	256
Cephalexin	0	1	3	9	11	33	41	20	13	5	3	0	2	2	8	32
Cefepime	60	0	35	26	14	3	0	1	0	0	1	0	0	3	0.5	2
Ceftiofur	22	52	39	12	4	4	3	0	1	1	1	2	1	1	0.25	4
Ciprofloxacin	2	16	23	23	20	18	23	12	4	1	0	0	0	1	2	16
Doxycycline	0	10	50	43	16	5	6	2	4	1	1	0	4	1	1	8
Enrofloxacin	10	5	25	23	24	19	19	11	7	0	0	0	0	0	2	16
Florfenicol	0	6	29	41	23	14	10	7	5	0	3	2	2	1	1	16
Gamithromycin	0	4	45	8	6	6	3	8	5	17	1	18	16	6	8	512
Gentamicin	1	0	0	8	25	30	32	21	11	3	2	1	5	4	8	64
Kanamycin	0	0	1	0	5	11	37	37	17	11	2	4	6	12	16	512
Lincomycin	0	1	1	6	10	32	33	25	11	5	5	2	6	6	8	256
Lomefloxacin	10	3	19	35	17	18	12	13	6	8	2	0	0	0	2	32
Oxacillin	0	5	13	8	29	20	25	21	3	3	5	1	5	5	4	128
Polymyxin B	0	50	49	21	1	2	2	3	0	4	1	2	5	3	0.5	64
Penicillin	0	10	52	27	19	7	1	4	7	2	0	5	0	9	1	256
Spectinomycin	0	11	9	18	20	16	20	21	15	4	2	2	3	2	4	32
Tulathromycin	0	1	9	23	24	26	24	9	11	6	2	0	3	5	4	64
Tildipirosin	0	11	34	30	22	10	9	4	4	1	0	1	10	7	1	512
Tetracycline	3	15	20	17	13	16	19	14	17	4	2	2	0	1	4	32
Tilmicosin	0	1	6	13	27	27	28	8	13	8	3	1	3	5	4	64

	Number of *H. parasuis* strains with MICs (μg/mL)															
	<0.06/1.19	0.06/1.19	0.12/2.38	0.25/4.75	0.5/9.5	1/19	2/38	4/76	8/152	16/304	32/608	64/1216	218/2432	>218/2432		
Trimethoprim/sulfamethoxazole	0	0	0	2	2	6	16	45	22	7	10	5	4	1	16	128

**Note:**

No CLSI breakpoints or other references available.

### Prevalence of biofilm formation and virulent characteristics

These isolates also displayed different capabilities for biofilm formation. A total of 99 isolates of *H. parasuis* (69.2%) were biofilm positive, and 59.6% (59/99) performed weak biofilm-forming ability. However, 24 isolates were moderate biofilm producers and 16 isolates (11.2 %) were strong biofilm producers. A total of 44 (30.8%) *H. parasuis* strains were not able to form biofilms on our polystyrene test surfaces (Table 4). The virulence characteristics were briefly defined according the serotype by previous report (Oliveira & Pijoan, 2004). The virulence characteristics for 143 *H. parasuis* were shown in Table 4. A total of 70 *H. parasuis* strains were highly virulent, 36 *H. parasuis* strains were moderately virulent and non-virulent *H. parasuis* were 26 strains.

**Table 4:** *H. parasuis* strains with differing biofilm profiles and virulent characteristics.

Biofilm-forming ability	Number of isolates	Involving serotypes	Virulent characteristics*		
			High	Moderate	Non-virulent
Strong	16	4,5,7,10,12,13, NT	10	3	2
Moderate	24	4,5,6,7,8,13,15, NT	10	8	5
Weak	59	1,2,3,4,5,6,7,9,11,12,13,14, NT	30	14	10
Absent	44	1,2,3,4,5,6,7,8,19,12,13,14, NT	20	11	9

**Notes:**

* Highly virulent; 1, 5, 10, 12, 13, 14 serotype; moderately virulent: 2, 4, 15 serotype; Non-virulent; 3, 6, 7, 8, 9, 11 serotype. Serovars used as an indicator of virulence are according to Oliveira & Pijoan (2004). The NT (non-typeable) were not divided into highly, moderately or nonvirulent.

### Enterobacterial repetitive intergenic consensus-polymerase chain reaction

**Figure 3:**
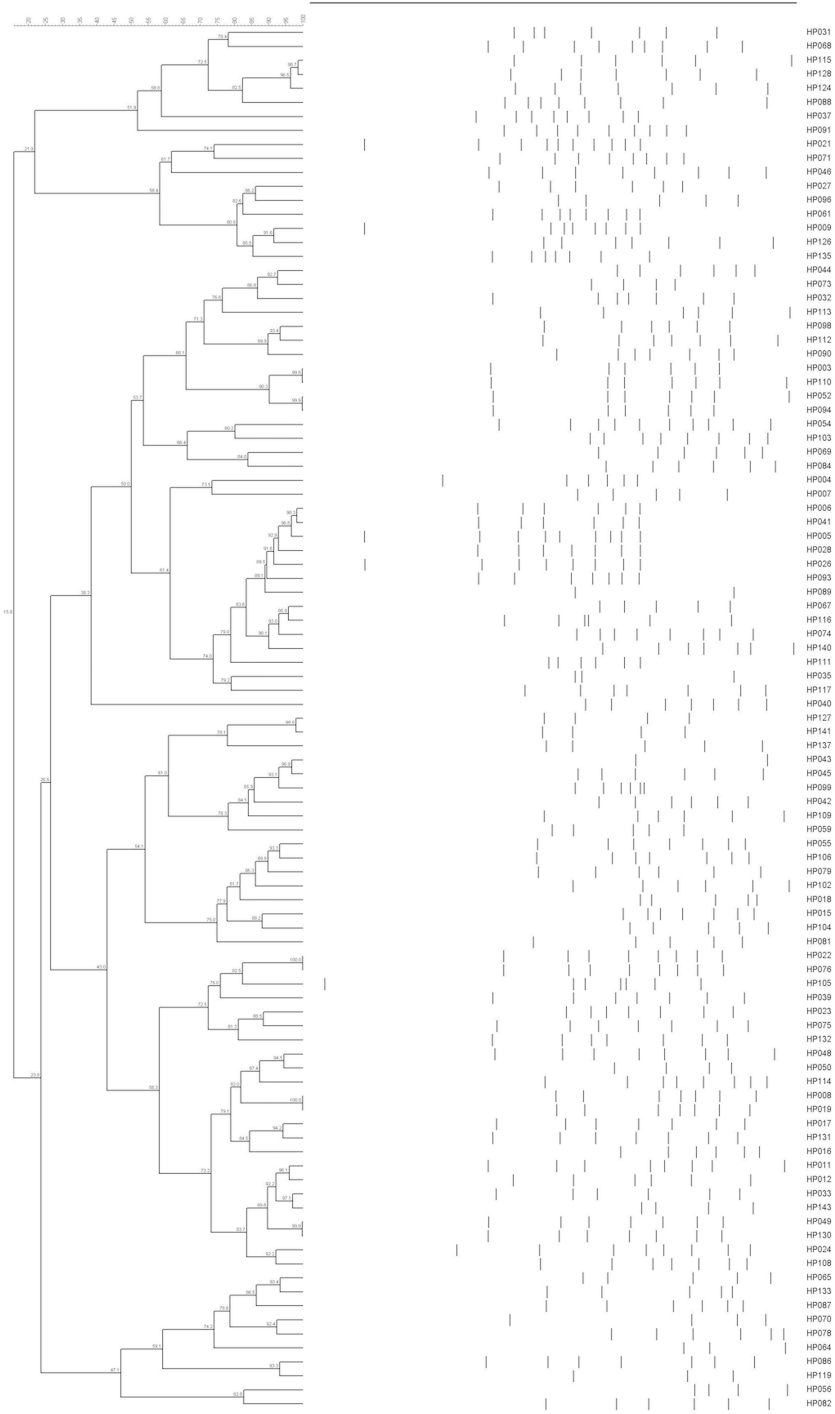
ERIC-PCR dendrogram of 99 *H. parasuis* field isolates.

All 143 *H. parasuis* strains were examined using ERIC with a 90% similarity cutoff. This analysis revealed 87 distinct groups that displayed heterogeneous ERIC patterns. Some isolates possessed 100% similarity with each other such as HP002 and HP048, HP008 and HP019, HP022 and HP076. Others such as HP049 and HP130 and five other pairs were almost perfect matches (Fig. 3). This indicated that the ERIC-PCR technique was more discriminative than serotyping and a broad genetic array was observed within serovars.

Among the 143 *H. parasuis* field isolates representing 15 serovars and nontypeable strains, 99 out of 143 strains were able to form some sort of biofilm. The distribution of this ability among the 15 serovars and nontypeable *H. parasuis* was diverse. Almost all the multiple antibiotic resistant isolates tested positive for biofilm formation. However, biofilm producers possessed higher resistance rates to more antimicrobials than non-producers. This included resistance to lincomycin, ampicillin, penicillin, gentamicin, kanamycin, florfenicol, polymyxin B, cephalexin, lomefloxacin, cefepime, tetracycline, tulathromycin, doxycycline, ceftiofur, amoxicillin, enrofloxacin, ciprofloxacin, and tilmicosin. Moreover, biofilm producers possessed lower resistance rates to trimethoprim/sulfamethoxazole, oxacillin, spectinomycin and when compared with non-biofilm producers (Table 5).

**Table 5:** Antimicrobial susceptibility of *H. parasuis* for biofilm positive isolates and biofilm negative isolates.

Antimicrobials	Biofilm positive isolates (*n* = 99)	Biofilm negative isolates (*n* = 44)
	Range of MIC (μg/mL)	Range of MIC (μg/mL)
Amoxicillin	0.25–64	0.25–16
Ampicillin	<0.25–>512	0.25–>512
Cephalexin	0.25–>512	1–128
Cefepime	<0.25–>512	<0.25–16
Ceftiofur	<0.25–>512	<0.25–8
Ciprofloxacin	<0.25–>512	0.25–16
Doxycycline	0.25–>512	0.25–32
Enrofloxacin	<0.25–32	<0.25–32
Florfenicol	0.25–>512	0.25–128
Gamithromycin	0.25–>512	0.5–>512
Gentamicin	<0.25–>512	1–512
Kanamycin	0.5–>512	2–512
Lincomycin	0.5–>512	0.25–>512
Lomefloxacin	<0.25–64	<0.25–64
Oxacillin	0.25–>512	0.25–>512
Polymyxin B	0.25–>512	0.25–512
Penicillin	0.25–>512	0.25–>512
Spectinomycin	0.25–>512	0.25–256
Tulathromycin	0.5–>512	0.25–>512
Tildipirosin	0.25–>512	0.25–>512
Tetracycline	<0.25–>512	0.25–256
Tilmicosin	0.5–>512	0.25–>128
Trimethoprim/sulfamethoxazole	0.5–>512	0.5–>512

Enterobacterial repetitive intergenic consensus-polymerase chain reaction was employed to evaluate the genetic relationship between the *H. parasuis* clinical strains with different biofilm-forming abilities. The 68 genotypes of the biofilm-forming isolates were heterogeneous and lacked any dominant clones so no correlation was identified (Fig. S1 is listed in Supplemental Material S2).

## Discussion

*Haemophilus parasuis* is one of the most common pathogen in pigs. However, the distribution and prevalence of serotypes and genotypes can vary considerably from region to region and over time within a given region. In this study, we analyzed serotypes, antibiotic resistance, biofilm formation and ERIC-PCR to characterize *H. parasuis* strains isolated from pigs.

Polymerase chain reaction technique is a rapid and specific method for the molecular serotyping, and had been used for *H. parasuis* serotyping (Jia et al., 2017; Lacouture et al., 2017; Wang et al., 2017). Accurate serotype identification is critical for epidemiological investigations or vaccine selection in *H. parasuis* infections. The indirect hemagglutinin assay is a good additional test for serotyping of *H. parasuis* if the GD methodology fails to type or shows unusual cross-reactions (Turni & Blackall, 2005). In this study, we used GD and IHA tests for serotyping. Global serological surveys of *H. parasuis* identified the most prevalent serotypes as 5, 4, 2, and 13 in Spain, 5, 4, and 13 in Denmark, 4, 5, 13, and 7 in North America, 1, 2, 4, 5, and 13 in the Netherlands and 4, 14, 5, 13, and 2 in Brazil and 4, 13, and 5 in Northern Italy (Angen, Svensmark & Mittal, 2004; Dijkman et al., 2012; Luppi et al., 2013; Moreno et al., 2011; Rubies et al., 1999; Tadjine et al., 2004). Epidemiological studies in China indicated that the prevalent serotypes were 4, 5, 13, 14, and 12 in 2005 and 4, 5, 12, and 13 in 2010 (Cai et al., 2005; Zhou et al., 2010). In southern China in 2011, the serotypes 4, 5, 13, 15, and 2 predominated (Zhang et al., 2011). Other three studies in China indicated the documented serotypes were 4, 5, 14, 13 in 2015 and 5, 4, 7, 13 in 2016 and 4, 5, 7, 1 in 2017 (Chen et al., 2015; Ma et al., 2016; Wang et al., 2017). In addition to these studies, high NT isolation rates (10–40%) were reported in all previously described studies, which maybe become a dominant serotype in Germany, USA/Canada, Denmark and Brazil (Angen, Svensmark & Mittal, 2004; Kielstein & Rapp-Gabrielson, 1992; Rapp-Gabrielson & Gabrielson, 1992), and even became the most serotype in Brazil (Macedo et al., 2011; Moreno et al., 2011). In this work, serotypes 5, 4, 7, and 13 were the most frequently detected and 8.4% were NT. These showed identical serotype profiles as a previous study reporting serotypes 4, 5, and 13 as dominant (Cai et al., 2005; Zhang et al., 2011).

Treatment of infections with *H. parasuis* commonly includes broad-spectrum antimicrobials (Aarestrup et al., 2004; de la Fuente et al., 2007; Wissing, Nicolet & Boerlin, 2001; Zhou et al., 2010). Nevertheless, the level of antimicrobial susceptibility varied a lot between different countries. In the present study, high MIC values were found for china *H. parasuis* strains. Therefore, preventive and therapeutic effects on porcine *H. parasuis* strains should no longer be expected from these antimicrobials. Tulathromycin was 100% susceptible to *H. parasuis* had been reported for 68 strains from EU 2009–2012, which MIC_90_ was 2 μg/mL (El Garch et al., 2016), while MIC_90_ was 64 μg/mL in this study. The sensitivities for the animal specific antimicrobials gamithromycin and tildipirosin (new macrolide antimicrobials for respiratory diseases) have not been reported until the current work. The high MIC values we found suggested that the use of these drugs should be regulated. Furthermore, these results indicated that there has been a rapid increase in the rate of resistance. This attributable at least in part to the use of antibiotic additives in animal feeds and the extensive use of antimicrobial agents in veterinary medicine. Additionally, there is a limit that there are currently no approved clinical breakpoints available for *H. parasuis*. Therefore, we should strictly control the use of antimicrobial agents in food animals and need a continuous surveillance of antimicrobial susceptibility to minimize the emergence of resistance in future. In a previous study, we have detected the antimicrobial resistance genes for the 143 *H. parasuis* (Zhao et al., 2018), combined with the antimicrobial susceptibility results in this study, demonstrated that the high MIC values of *H. parasuis* in piglets is a combination of transferable antibiotic resistance genes and multiple target gene mutations.

Biofilm formation is essential for persistent infections (Costerton, Stewart & Greenberg, 1999). These structures can withstand host immune pressures and biofilm-producing strains are less susceptible to antimicrobials than biofilm-negative strains (Anderson & O’Toole, 2008; Hall-Stoodley & Stoodley, 2009; Zhang et al., 2014). *H. parasuis* forms biofilms in vitro and suggests a causal relationship between colonizers and biofilm growth (Jin et al., 2006). Individual *H. parasuis* isolates differed in their ability to form biofilms as has been reported previously (Zhang et al., 2014). Different serovars possessed different abilities to form biofilms and we confirmed a positive correlation between biofilm presence and antibiotic resistance especially with β-lactam antimicrobials (Moleres et al., 2015; Zhang et al., 2014).

In general, the 15 different serovars of *H. parasuis* could be divided into those that were highly virulent, moderately virulent and non-virulent although there may be individual variations (Oliveira & Pijoan, 2004). However, the high-biofilm production phenotype is not always being linked to virulence (Bello-Orti et al., 2014). It is worth noting that a recent study on the human pathogen *Streptococcus pneumoniae* showed that biofilm formation in vivo was associated with reduced invasiveness and a dampened cytokine response (Blanchette-Cain et al., 2013). In the present study, we detected significantly higher resistance levels among biofilm producers compared with non-biofilm producers. We also confirmed a positive correlation between biofilm presence and antibiotic resistance except for oxacillin, spectinomycin and trimethoprim/sulfamethoxazole. Biofilm-forming *H. parasuis* isolates have significantly higher resistance to β-lactams than the non-producers (Zhang et al., 2014). The persistence of antibiotic resistance within biofilms is another aspect that should not be neglected because it has a potential impact on both animal and public health.

In the current study, we also utilized ERIC-PCR and found it possessed discriminatory power for *H. parasuis*. ERIC-PCR has been used to successfully subtype *H. parasuis* strains isolated from different regions, confirming the high heterogeneity and high genetic variability of *H. parasuis* (Macedo et al., 2011; Oliveira et al., 2004; Olvera, Calsamiglia & Aragon, 2006; Rafiee et al., 2000). We obtained 87 genotypes among the 143 *H. parasuis* strains and 68 genotypes in the 99 biofilm producers similar to previous results (Zhang et al., 2011). This analysis demonstrated that there were no dominant clones. Comparisons of the ERIC-PCR results of the biofilm positive strains yielded no defined correlations between serovar and ERIC-PCR profile. No correlations between biofilm formation and serovar were found in this study.

## Conclusion

In conclusion, this study showed *H. parasuis* strains had high-MIC values to common antimicrobial agents in China. These showed a marked geographic variability indicating that the prudent use of antimicrobials is very important. These *H. parasuis* isolates were also diverse serologically and genetically and possessed with different levels of biofilm-forming ability in vitro. Biofilm formation correlated with their antimicrobial susceptibility. This study suggests the need for a continuous surveillance of clinical isolates of *H. parasuis*.

## Supplemental Information

10.7717/peerj.5040/supp-1 Supplemental Information 1 MICs of the 23 antimicrobials against 143 isolates of *H. parasuis*.

10.7717/peerj.5040/supp-2 Supplemental Information 2 Fig. S1. ERIC-PCR dendrogram of 143 *H. parasuis*.

## Additional Information and Declarations

### Data Availability

The following information was supplied regarding data availability: The raw data are provided in a Supplemental File.
